# Patellar and Achilles Tendon Thickness Differences among Athletes with Different Numbers of Meals per Day: A Cross-Sectional Study

**DOI:** 10.3390/ijerph19042468

**Published:** 2022-02-21

**Authors:** Santiago Navarro-Ledesma, Gabriel Gijon-Nogueron, Inmaculada Reina-Martín, Ana Belen Ortega-Avila, Leo Pruimboom

**Affiliations:** 1 Department of Physiotherapy, Faculty of Health Sciences, Campus of Melilla, University of Granada, Querol Street, 5, 52004 Melill, Spain; 2 Department of Nursing and Podiatry, Faculty of Health Sciences, University of Malaga, 3, Arquitecto Francisco Penalosa, 29002 Malaga, Spain; gagijon@uma.es (G.G.-N.); ireinamartin@gmail.com (I.R.-M.); anaortavi@uma.es (A.B.O.-A.); 3 PNI Europe, 2518 JP The Hague, The Netherlands; cpni.pruimboom@icloud.com

**Keywords:** Achilles tendon, patellar tendon, ultrasonography, runners, metabolic health, metabolic education

## Abstract

The objective of this study is to analyse differences in the thickness of the patellar (PT) and Achilles tendons (AT) among athletes with different number of meals per day. The design is a cross-sectional, observational study. A total of thirty-six male athletes (with mean age groups ranging from 31 to 40) were recruited and divided into three groups based on the number of daily meals they had (3, 4 or 5 meals). PT and AT were assessed by ultrasound. There were statistically significant differences in PT when comparing groups 1 and 3, at both longitudinal (*p* < 0.03) and transversal (*p* < 0.002) planes. There were no differences when comparing groups 1 and 2 or groups 2 and 3. There was a negative correlation between the number of meals per day and tendon thicknesses in both PT (longitudinal plane: r = −0.384; *p* = 0.02/transversal plane: r = −0.406; *p* = 0.01) and AT (transversal plane: r = −0.386; *p* = 0.02). In conclusion, there were patellar tendon thickness differences between participants and the number of daily meals could play a key role in tendon thickness, healing and performance.

## 1. Introduction

The Achilles (AT) and patellar (PT) tendons are commonly altered among athletes, and particularly in runners [1]. Running is one of the most popular sports activities and therefore the number of studies focus on runners and related injuries are increasing [2,3].

The overall incidence of injury to runners ranges from 18.2% to 94.4% [4]. The Achilles tendinitis present has an incidence of9.1–10.9% in runners, and patellar tendinitis has an incidence of 5.5–22.7%, being among the most frequent injuries in sports [4,5]. It is thought that high loading rates, when combined with suboptimal technique, may produce aberrant strain to lower extremity tendons, contributing to microtrauma that may trigger tendinopathies [6,7]. Furthermore, tendinopathy and tendon rupture are associated with obesity and associated metabolic conditions, such as insulin resistance and dyslipidaemia [8].

Ultrasound imaging (US) is frequently used to assess tendon morphology, and it is considered to be as reliable as magnetic resonance imaging [9]. The normal ultrasound characteristic of PT and AT is well established in a healthy population, but not in athletes; nevertheless, several abnormalities have been shown to be common in both populations [6].

Tendon changes can be diagnosed before they become symptomatic with US, possibly being reflective of adaptations or maladaptations [10,11]. Additionally, identifying other factors that may produce tendon changes would help in detecting presymptomatic athletes and would enable the introduction of modifications to their training regimen [6,12]. One of these factors could be the number of meals per day in runners. In this regard, there is evidence showing that high levels of glucose provoke higher insulin levels and a consecutive glucose disbalance. Hypoglycaemia can induce a rebound effect on the level of gluconeogenesis induced by glucocorticoids, which in excess may affect tendon structure [8]. Glucocorticoid release is regulated by the hypothalamic–pituitary–adrenal (HPA) axis, and its function can be summarized in two ways. First, an acute activation of the HPA axis generates a glucocorticoid peak, which increases gluconeogenesis in the liver and inhibits insulin production [13]. If the subject presents proper energy reserve and distribution, they will be able to respond adequately to the stress factors which caused the stress response and activation of the sympathetic nervous system and the HPA axis [14]. Second, when the HPA axis loses its rhythm and is chronically activated, cortisol release is constant, and this could provoke maladaptive responses leading to insulin resistance, a condition that could cause an increase in and the acceleration of the generation of cross links [15]. The opposite is also a possible cause of collagen tissue atrophy and possible injuries. Insulin resistance and HYPER-insulinemia induce the overexpression of undercarboxylated osteocalcin and loss of bone density and collagen tissue damage by activation of RANKL in bones and increased uptake of glucose in collagen tissue, respectively [16]. The increase in intracellular glucose activates the polyol pathway that induces the production of sorbitol and fructose under severe oxidative stress [17]. The latter could be responsible for mitochondrial damage and tissue breakdown [17].

The composition of food has been extensively researched and understood. However, meal frequency and timing are important aspects of nutrition, and how they impact health, performance and healing needs to be further investigated [18]. To the best of our knowledge, there have not been studies investigating to what extent and if the number of meals per day correlate with PT and AT thicknesses in runners. Our hypothesis is that differences in the number of meals per day could explain morphological changes in tendons, being the trigger factor which leads to a metabolic disorder. Hence, the aim of the study was to analyse differences in PT and AT thicknesses between groups with different meal frequencies (3,4 or 5). The second outcome is to study if meal frequency could be related with thickness of the PT and AT.

## 2. Material and Methods

This study was conducted in full accordance with the provisions of the Declaration of Helsinki regarding ethical principles for medical research involving human subjects, and was approved by the Medical Ethics Committee of the University of Malaga (CEUMA122017H), Spain.

This was a cross-sectional, observational study. We used the STROBE-statement, which was developed to scientifically strengthen the impact of observational studies in epidemiology [19].

A total of thirty-six male athletes were recruited from different athletic clubs and volunteered to participate in our study. All subjects were at least 18 years old and were able to follow the study instructions. Informed consent was obtained in every case.

The inclusion criteria were:

(1) The runners had run at least 50 km per week during the previous six consecutive months, (2) at a pace of no more than six minutes per km [20];

The exclusion criteria were:

(1) osteo-degenerative disease, (2) metabolic disease, (3) neurological problems or surgical intervention in the lower limb, (4) processes of an infectious, (5) cancer, (6) cognitive impairment, (7) musculoskeletal injuries of the lower limbs in the last three months, and (8) use of corticosteroids.

### 2.1. Sample Size

The sample size was determined by application of the EPIDAT program, using the criterion of AT with a detectable difference of the mean of 0.818 to evaluate the statistical power [21]. The study was designed to detect changes exceeding 0.8 (large effect size) for a variation of the sample according to the above classification, with a type I error of 0.05 and a type II error of 0.2. This calculation produced a necessary sample size of 18 subjects in each group.

### 2.2. Study Protocol

The subjects were evaluated at the podiatric healthcare teaching unit of the University. In a single session, the relevant study data were obtained—age, BMI, and number of meals per day, together with the ultrasound measurements, performed by an expert in musculoskeletal ultrasound imaging with 8 years of experience and a second examiner assistant, who placed a shield on the ultrasound screen to assure the blinding, taking all the data from the screen. The participants were divided into three groups, based on the number of meals per day: Group 1: 3 meals; Group 2: 4 meals; Group 3: 5 meals. The intraclass correlation coefficient of ultrasound examiner was used with 10 participants to evaluate the reproducibility of measurement of the thickness and cross-sectional area of the AT and the thickness of the AT and PT, with a sample of ten subjects measured at baseline and after 24 h. They were estimated by calculating the ICC for the second author, using a one-way random effect model, and found to be excellent (ICC 0.94: 95% CI: 0.90–0.96) at 0, and 0.87 (95% CI: 0.80–0.92) at 60.

### 2.3. Ultrasound Measurements

A diagnostic ultrasound unit, Sonosite M-turbo (GE Healthcare, Wauwatosa, WI, USA) with a dynamic range of up to 165 dB, was used. In addition, a 6–13 MHz linear transducer with 196 piezoelectric crystals fitted with a specific ultrasound system, SonoMB^®^ multi-beam imaging, was employed to increase resolution and improve the visualisation of subtle physiological and tissue differences. These images were captured as grey-scale images with 256 levels. All participants received standardised ultrasonography in the dominant lower limb. The ultrasound images were obtained by a single examiner, a qualified physiotherapist with 9 years of experience with musculoskeletal ultrasound imaging. The ultrasound examiner was blind to the characteristics of the participants. Simultaneously, the data were recorded by a research assistant, who was blind to the characteristics. Each participant was issued an identification number, which was the only information provided to the examiners. All ultrasound measures are expressed in millimetres [22].

### 2.4. Achilles Tendon

**Figure 1:**
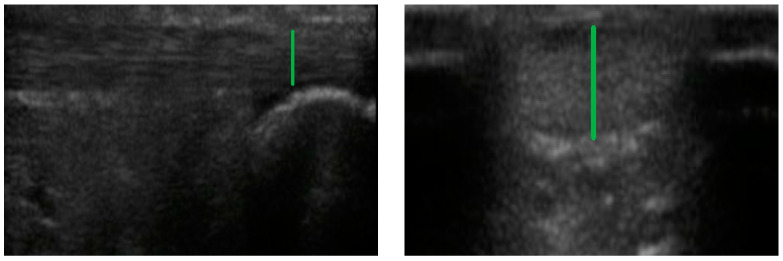
AT measurement in longitudinal (**left**) and transversal (**right**) axis.

The participants adopted a prone position, and the AT was scanned both longitudinally and transversely, with the transducer placed on the AT. The thickness of the tendon was measured at the level of the medial malleolus, in order to standardise the measurements, as was shown in previous studies [23]. The thickness of the AT was measured by its maximum anteroposterior diameter (Figure 1) [22].

### 2.5. Patellar Tendon

**Figure 2:**
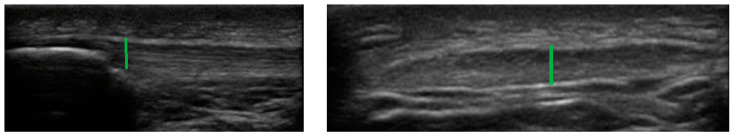
PT measurement in longitudinal (**left**) and transversal (**right**) axis.

The participants adopted a supine position. The placement of the transductor and subsequently the measurements were taken 2 cm distal to the end of the patella, and both longitudinal and transversal axes were measured [24]. The thickness of the PT was measured by its maximum anteroposterior diameter (Figure 2) [22].

### 2.6. Statistical Analysis

Normality for all US variables was explored using the Shapiro–Wilk test for the three groups of participants. To determine between-groups differences for all the outcome measurements, one-way ANOVA test was calculated with Tukey post-hoc estimation [22]. A *p*-value less than 0.05 was considered statistically significant. To calculate the intra-rater reliability of all the US variables, three measurements of each one were collected, and a two-way mixed (3,1), consistency, intraclass correlation coefficient (ICC) was then calculated. A reliability coefficient < 0.50 was an indication of “poor” reliability; “moderate” between 0.50 and 0.75; “good” between 0.76 and 0.90; and “excellent” over 0.90 [25]. The standard error of measurement (SEM) and the minimal detectable change at 95% confidence of interval (MCD95) were also obtained. To determine the correlations between tendon thicknesses (PT and AT) and the number of meals per day, a Pearson correlation coefficient was calculated for a normal data distribution, or a Spearman’s coefficient in the case of absence of normality. Weak correlation was defined as values between 0.3 and 0.5; between 0.5 and 0.7 correlation was considered moderate; and strong was considered greater than 0.7.

For all the US calculations, three measurements were taken by the examiner, and an average of three was used for the statistical analysis for each angle. An interval of 1 min was provided between measures, and the patient was encouraged to move freely. Patients were then repositioned and the second and third sets of measurements were successively taken.

## 3. Results

**Figure 3:**
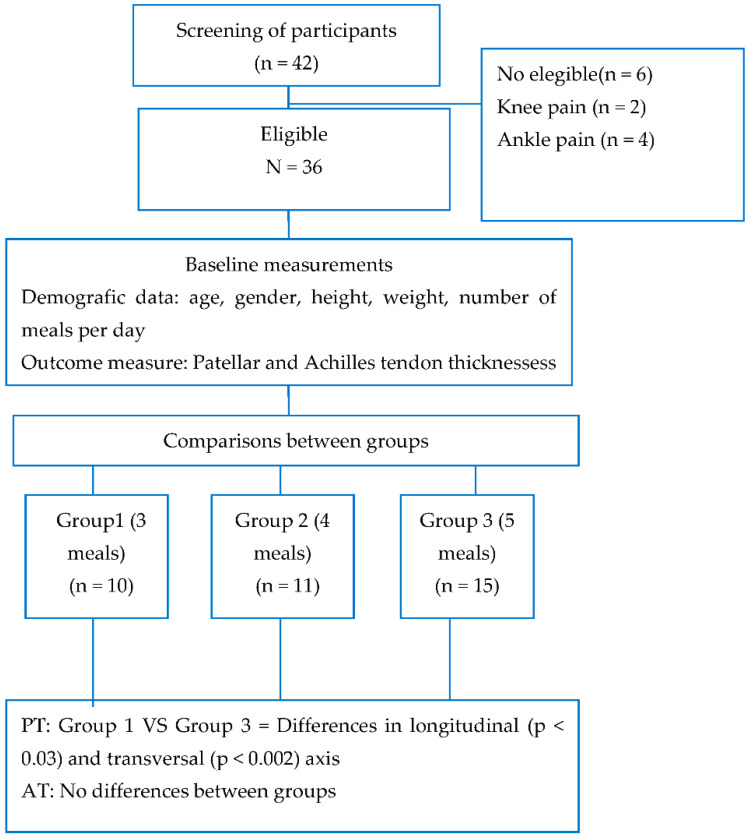
Low chart of participants.

The recruitment included a total of 42 participants, with six participants who did not fit the inclusion criteria because of the presence of pain. A final number of 36 participants were enrolled in the study (see flow Figure 3).

### Sample Characteristics

Demographic characteristics are shown in Table 1. There were not significant differences between groups in terms of age, BMI, number of kilometres per week and number of training hours per week.

Mean values for the outcome measures and intra-rater reliability data: Mean values of PT and AT (at both longitudinal and transversal planes, expressed in millimetres), for all the groups are presented in Table 2. There were statistically significant differences in PT when comparing the three groups, while no differences were found for the AT measurements.

Differences in patellar and Achilles tendon thicknesses between groups: comparisons between groups are described in detail in Table 3. There were statistically significant differences in PT at both longitudinal and transversal planes between group 1 and group 3. There were no statistically significant differences between groups for AT at both longitudinal and transversal axis.

Association between patellar and Achilles tendon thicknesses and the number of meals per day: correlations between tendon thicknesses (PT and AT at both longitudinal and transversal planes) and the number of meals per day are shown in Table 4. There was a negative correlation between the number of meals per day and tendon thicknesses in both PT (longitudinal plane: r = −0.384; *p* = 0.02/transversal plane: r = −0.406; *p* = 0.01) and AT (r = −0.386, *p* = 0.02), showing that in these parameters there is a tendency to present thinner tendons when the number of meals increase.

## 4. Discussion

The aim of the present study was to study differences in PT and AT thicknesses between groups with different number of meal frequency per day (3,4 or 5 meals). Secondary, to analyse the correlation between the thickness of the PT and AT and the different number of meals per day among athletes (3, 4 or 5 meals).

There were statistically significant differences in the PT when comparing groups 1 and 3, at both longitudinal (*p* < 0.03) and transversal (*p* < 0.002) planes. There were no differences when comparing groups 1 and 2 or groups 2 and 3. Moreover, there was a negative correlation between the number of meals per day and the tendon thicknesses in both PT (longitudinal planes−0.384; *p* = 0.02/transversal planes: r = −0.406; *p* = 0.01) and AT (r = −0.386, *p* = 0.02). There were no significant differences in AT when comparing the three groups.

Comparisons with other studies are difficult because this is the first study analysing differences in tendon thicknesses among athletes in relationship with the number of meals per day as the main responsible factor. Our AT thickness measures are in line with those reported in previous studies with athletes, as well as with those related to PT thickness. Currently, a certain minimum level of load is thought to be necessary to provoke a change in the tendon [26]. Endurance exercise puts a high burden on several tissues, including tendons, cartilage, bone and even the brain [27]. Our results show that reducing meal frequency could protect several tissues for breakdown when athletes engage in endurance sports. Several hypotheses related with the benefit of a low meal frequency versus a high meal frequency (3 meals/day versus 4 or 5 meals/day) include several immunological and metabolic pathways:

1. Postprandial inflammatory response: Postprandial inflammation can put a high burden on energy availability for the whole body, including the brain. Furthermore, it increases with meal size, meal frequency and consumption of foul food, exhibiting the metabolic conflict between the immune system and the brain [28].

The SLC2A1 gene is responsible for the production of GLUT1 glucose transporters andSLC2A4 gene expression for GLUT4 glucose transporter in muscle. Under physiological circumstances, the brain would be allowed more energy availability given the increase in SLC2A1 expression in the brain and a decrease in SLC2A4 in skeletal muscle when a certain circulating glucose concentration appears, with both energy-demanding tissues working synergistically to change the distribution of glucose [28].

However, a prolonged activation of the immune system, as may be present in those with higher numbers of meals per day, allocates glucose chronically to the immune system through immune-controlled downregulation of GLUT1 transporters at the level of the blood–brain barrier and a reduction of GLUT4 transporters at the level of muscle and adipose tissue [29,30]. This selfish behaviour of the immune system is responsible for the majority of chronic diseases [31].

2. Insulin resistance, high blood sugar and the polyol pathway: Insulin-resistance and altered glucose metabolism are the distinguishing characteristics of type-2 diabetes and are also some of the symptoms associated with metabolic disorders. In relation to this, it is known that the generation of uric acid during fructose metabolism results in mitochondrial oxidative stress and an impairment in ATP production [32]. High glycaemic diets in those presenting insulin resistance may increase the polyol pathway, which results in the conversion of glucose to sorbitol by aldose reductase (AR), followed by the conversion of sorbitol to fructose by sorbitol dehydrogenase (SDH), which would result in endogenous fructose production [33,34]. Uric acid has been shown to present functions as an anti-oxidant extracellularly; nevertheless, many studies show uric acid can induce both cytosolic and mitochondrial oxidative stress via the induction of NADPH oxidase, and possibly by direct effects [32,35,36]. Therefore, strategies to decrease AR levels may be indicated and would protect from acid uric metabolic disorders [37].

Furthermore, the overexpression of undercarboxylated osteocalcin in a possible state of insulin resistance combined with hyper-insulinemia also produce loss of bone density and collagen tissue damage by activation of RANKL in bones and increased uptake of glucose in collagen tissue, respectively [16].

3. Energy distribution disorder (disposal soma): The brain and the immune system are favoured energetically when the immune system is activated through danger signals. However, a chronic allocation of glucose to the immune system has been observed in people with chronic inflammatory states given the prolonged activation of the immune system. This has been described through immune-controlled downregulation of GLUT1 transporters at the level of the blood–brain barrier and a reduction of GLUT4 transporters at both muscle and adipose tissue [29,30]. In this regard, most, if not all chronic diseases may be the consequence of the so-called selfish immune system behaviour [31].

This study presents strengths because it is the first showing differences in PT and AT thicknesses between groups of athletes with different number of meals per day. Similar demographic characteristics of different groups were presented. Moreover, intra-rater reliability was excellent for the ultrasound measurements, and a careful screening for exclusion criteria was conducted by an expert using US imaging during both assessment and the tendon thickness measurements. On the other hand, some limitations should be recognized. First, the relatively small sample size of this study indicates the need to interpret the results with caution. Second, age differences between groups may also influence tendon thickness differences, as well as other residual cofounding biases such as the composition of meals, sleep or strength training. Finally, other factors such as insulin resistance or glucose blood levels, that have shown their association with chronic disorders, were not assessed [31]. Future research in this field addressing these limitations is needed.

The extrapolation of the presented results can be of huge relevance because they may prevent changes in tendons and metabolic dysfunctions, which lead to mitochondrial damage and tissue breakdown. Hence, new approaches to optimize endurance training and avoid injuries based on the reduction of meals per day are proposed in addition to those existing [38].

In addition, although not significantly, our results showed that there was a trend in which athletes consuming less meals per day exhibited a higher number of training kilometres per week and less time spent on it. This is also of interest because it may elucidate another way of increasing performance. Therefore, a diet based on few meals per day may decrease tissue damage in runners. However, this is only a hypothesis and future studies are required to confirm it.

## 5. Conclusions

This study shows preliminary evidence that runners with higher meals per day present thinning changes in the PT and AT thicknesses. Longitudinal studies, including the assessment of insulin and glucose blood levels, should be developed to corroborate our findings.

## Figures and Tables

**Table 1:** Demographic characteristics, mean (SD).

	Group 1 (3 Meals/Day) N = 10	Group 2 (4 Meals/Day) N = 11	Group 3 (5 Meals/Day) N = 15
Age (years; SD)	40.5 (8.29)	34.9 (9.35)	31.6 (10.6)
Body mass index (SD)	24.2 (2.26)	23.3 (2.82)	23 (3.59)
Number of kms/week (SD)	58 (27.4)	57.6 (30.3)	53.1 (20.6)
Number of training hours/week (SD)	10.1	10.2	12.5

SD: Standard Deviation.

**Table 2:** Mean values (95% CI) of Patellar tendon (PT) and Achilles Tendon (AT) both at longitudinal and transversal view expressed in millimetres in different groups; F: one-factor ANOVA for differences between groups.

	Group 1	Group 2	Group 3	F	*p* Values
PT (longitudinal)	5.70 (4.10–7.40)	4.91 (3.20–7.10)	4.69 (3.40–6.00)	3.659	0.047 *
PT (transversal)	4.34 (3.70–5.70)	4.10 (2.90–7.00)	3.68 (2.90–4.90)	4.022	0.036 *
AT (longitudinal)	21.4 (17–28)	22.2 (19.1–25.5)	22.2 (18.7–27.6)	0.234	0.793
AT (transversal)	5.60 (4.20–7.20)	5.00 (4.30–7.50)	4.60 (3.30–6.10)	2.986	0.073

* Statistically significant (*p* < 0.05).

**Table 3:** Between groups differences in patellar tendon (PT) and Achilles tendon (AT).

	Group 1 vs. Group 2	*p* Values	Group 1 vs. Group 3	*p* Values	Group 2 vs. Group 3	*p* Values
PT (longitudinal) ^a^	0.791 ^b^	0.239	1.007 ^b^	0.038 *	0.216 ^b^	0.844
PT (transversal) ^a^	0.240 ^b^	0.832	0.660 ^b^	0.028 *	0.420 ^b^	0.546
AT (longitudinal) ^b^	−0.831 ^a^	0.780	−0.833 ^a^	0.751	−0.002 ^a^	1
AT (transversal) ^b^	0.317 ^a^	0.701	0.863 ^a^	0.062	0.546 ^a^	0.290

*: Statistically significant (*p* < 0.05). ^a^: Tukey Post-Hoc test was carried out. ^b^: Games–Howell Post-Hoc test was carried out.

**Table 4:** Correlations between patellar (PT) and Achilles tendon (AT) thicknesses (at both longitudinal and transversal planes) and the number of meals per day.

	Number of Meals Per Day
PT (longitudinal) ^†^ (r)	−0.384 *p* = 0.02 *
PT (transversal) ^†^ (r)	−0.406 *p* = 0.01 *
AT (longitudinal) ^††^ (r)	0.118 *p* = 0.494
AT (transversal) ^††^ (r)	−0.386 *p* = 0.02 *

^†^: Pearson correlation coefficient was calculated. ^††^: Spearman correlation coefficient was calculated. *: Statistically significant (*p* < 0.05).

## Abbreviations

Achilles Tendon	(AT)
Patellar tendon	(PT)
Ultrasound imaging	(US)
Hypothalamic–pituitary–adrenal	(HPA)
